# Factor Structure and Psychometric Properties of the Family Quality of Life Questionnaire for Children With Developmental Disabilities in China

**DOI:** 10.3389/fpsyg.2020.01585

**Published:** 2020-08-26

**Authors:** Rujun Huang, Renhong Shen, Su Qiong Xu

**Affiliations:** ^1^Guizhou University of Engineering Science, Bijie, China; ^2^College of Education Science, Chongqing Normal University, Chongqing, China; ^3^The Key Laboratory for Diagnosis and Educational Technology of Children with Special Education Needs, Chongqing, China

**Keywords:** children with developmental disabilities, Quality of Life, factor structure, psychometric properties, China

## Abstract

The purpose of this study was to explore the factor structure and psychometric properties of the Chinese version of Family Quality of Life Questionnaire (Chinese FQoL-Q) for children with developmental disabilities (CDD) under the background of Chinese culture. The item analysis, exploratory factor analysis, confirmatory factor analysis, and reliability test were carried out on survey data from a sample of 845 families of CDD. It was found that the Chinese FQoL-Q involved seven factors with 35 items, including economy and leisure, physical and mental health, parenting, family communication, support from others, professional support, and career development. The measurement model of Chinese FQoL-Q reflected traditional Chinese culture and parents’ perception of education or rehabilitation for CDD in China. The Chinese FQoL-Q is reliable and valid for assessing the quality of life for family members with developmental disabilities.

## Introduction

Since the 1980s, research on quality of life (QoL) for individuals in relation to special educational needs has gradually emerged and obtained fruitful achievements, in order to satisfy the increasing demands on welfare policy-making, supportive project-design, and service evaluation for people with disabilities (Schalock et al., 2002). At the beginning of the 21st century, the research focus of QoL shifted from an individual perspective toward family, as family has gradually become the main source of support for their children. Legislation and policies in countries (i.e., *Individuals with Disabilities Education Act* in the United States and *Persons with Disabilities Education Ordinance* in China) have also attached great significance to the role of family for education and rehabilitation of their family members with disabilities (Zuna et al., 2009). Family quality of life (FQoL) has thus become an area of considerable interest internationally (e.g., Brown et al., 2006).

The concept of FQoL was defined as “conditions where the family’s needs are met, and family members enjoy their life together as a family and have the chance to do things which are important to them” (Park et al., 2002). This definition has been challenged due to the dynamic sense of the family’s well-being, in which context, system, policies, and programs interact on both individual and family level needs (Zuna et al., 2010). Such an interactive perspective of FQoL might be related to the adoption of systemic and ecological models of human development, and emphasize on examining family members’ perceptions and dynamics of the family unit as a whole. Zuna et al. (2009) further proposed four major explanatory concepts to build FQoL theory: (1) systemic concepts, (2) performance concepts, (3) individual-member concepts, and (4) family unit concepts. These factors singly or collectively interact with each other to result in an FQoL outcome, which is conducive to improving practices and services for families of, and individuals with, disabilities. Except for the four concepts, Zuna et al. (2010) also stressed three themes in relation to FQoL: (1) the subjective impressions of family members’ satisfaction, (2)meeting individual family member’s needs, and (3) the family as a unit in evaluating FQoL. In general, the complexity of FQoL implies that the development of measurement tools has been a challenge, especially when people with disabilities are involved.

In a western context, there are three important disability-related measures of FQoL (Zuna et al., 2009). The first one is the Beach Center Family Quality of Life Scale (Beach Center FQoL-S; Hoffman et al., 2006), which has been adapted and validated in different languages and regions (e.g., Balcells-Balcells et al., 2011; Chiu et al., 2017). It is a 5-point Likert scale and is composed of 25 items on five dimensions including physical/material well-being, family interaction, parenting, disability-related support, and emotional well-being. The second important disability-related measurement is the International Family Quality of Life Survey, which evaluates FQoL of individuals with disabilities of any age and has been translated into 16 languages and widely used in over 20 countries (International FQoL-S; Brown et al., 2006; Isaacs et al., 2007). It measures FQoL based on nine dimensions (health, financial wellbeing, family relationships, support from other people, disability-related services, influence of values, careers and preparing for careers, leisure and recreation, and community interaction) from six aspects (importance, opportunities, initiative, stability, attainment, and satisfaction). It is also a 5-point Likert scale, and families can add narrative description. The International FQoL-S was revised in 2007 and its confirmatory study on the factorial structure is still in progress (Isaacs et al., 2012). The third instrument is the Latin American Family Quality of Life Scale (Latin American FQoL-S) that was formulated based on 183 families of children with intellectual disabilities in Latin America. This scale was a Spanish-language scale, and consists of 43 items on six dimensions: emotional well-being, personal strength and development, rules of cohabitation, physical/material well-being, family life, and interpersonal and community relations (Aznar and Castanon, 2005).

The Beach Center FQoL-S and the International FQoL-S are more widely used (Samuel et al., 2012), whereas the Latin American FQoL-S was specially formulated on the basis of the high unemployment rate and poverty status in Latin America. There are some differences among these scales. For example, despite both the Beach Center FQoL-S and the Latin American FQoL-S having dimensions of emotional well-being and physical/material well-being, the specific items on the two dimensions are different. Compared to the Beach Center FQoL-S, the Latin American FQoL-S has a special item “*live in peace*” on its emotional well-being dimension. This might be related to the colonial history of Latin America (Martin and Wasserman, 2007, translated by Huang, 2007), during which their policies were not stable (Ministry of Business, 2017) and the Latin Americans were longing for peace (Su, 1988). That also explains why the Latin American FQoL-S focuses on items like “*eat, cloth*” on physical/material well-being, which is consistent with Latin American’s poor economy, high unemployment rate, and unfair distribution (Aznar and Castanon, 2005; Wu, 2014). On the contrary, the Beach Center FQoL-S focuses on items such as “*My family members have transportation to get to the places they need to be*” on physical/material well-being, probably because of relatively good economic condition in the United States.

A common concern underlying these differences among the instruments is related to culture. That is, the present instruments did not take enough consideration on special populations such as groups with low income or minorities (Hoffman et al., 2006). It is thus imperative to examine the quality of life based on countries, language, and cultural background (Verdugo et al., 2005).

In China, an individual submits to his or her family and family interests are more important than the individual’s (Xu et al., 2018). Compared to the western culture that emphasizes individualism, the Chinese culture tends to attach more significance to family, the collective, and the country (Huo, 2018). Moreover, the fact that the development of special education in China started later than in Western countries has resulted in a lack of diversity and foresight in parents’ perception of education, rehabilitation, and demand for professional support services. Due to collectivism and the limited development of special education in China, Chinese parents are likely to suppress their own needs, and focus more on the defects of their children and family difficulties. In this regard, the Chinese parents’ demand for professional support services may be different from that in Western countries. These cultural differences probably lead to different factorial structures of FQoL of children with developmental disabilities (CDD) between China and the West.

Domestic research on FQoL of CDD mainly focuses on investigating the *status quo* by borrowing the Western scales (Hu and Wang, 2012) or using individual interviews (Luo, 2014; Li, 2016). It is necessary to construct a FQoL scale for families of CDD in China. The theoretical consideration of this study would first reference four major explanatory concepts of FQoL theory proposed by Zuna et al. (2009), and three themes of FQoL (Zuna et al., 2010) to construct initial interview questions in relation to family relationships, access to information and services, child functioning, and overall life situation. The second theoretical support comes from the three most important disability-related measures of FQoL. The dimensions and items on the Western FQoL scales are crucial references for constructing a Chinese version of FQoL. By doing so, the cultural differences emerge as the third theoretical consideration for the current study. The fourth theoretical consideration of the current study is related to needs and dilemmas that the Chinese family of CDD encounter and suffer from. Domestic research has indicated three major dilemmas: financial difficulty, poor quality of professional rehabilitation, and shortage of time and energy in taking good care of their kids (Huang and Liu, 2006; Chen and Chen, 2016). Based on the four theoretical considerations, this study aims at constructing factor structure and measurement index systems of FQoL of CDD in China, in order to better evaluate the quality of family support, and to better design and implement individualized family support programs.

## Materials and Methods

### Participants

Convenient sampling of 845 parents of CDD were recruited from special education schools, disabled persons’ federations, and private institutions in Henan province (middle part of China), Chongqing City and Sichuan province (southwest of China), and Guangdong province (southeast of China). It is worth noting that the CDD in this study refers to individuals under the age of 18 who have been medically diagnosed with significant and long-term developmental delays due to physical or psychological reasons. Among 845 participants, 272 reported having only one child (32.2%), 564 two or more children (66.7%) in their families, and nine did not report the number of their children; 270 were males (32.0%), 570 females(67.5%), and five did not report their gender; 375 were from the city(44.4%), 400 from the countryside(47.3%), and 70 did not report their location; 550 were mothers(65.1%), 240 fathers(28.4%), 34 parental grandparents(4%), 17 maternal grandparents (2.0%), and four did not report their identification; 369 residential conditions involved a nuclear family (43.7%), 355 were three generations under one roof (42%), 62 were extended family (7.3%), 45 were single-parents family (5.3%), four were four generations under one roof (0.5%), and 10 did not report their residential condition.

### Procedure

The research process references the relational ethical framework proposed by Flinders (1992), and is in line with ethical norms from the principles of informed consent, confidentiality, and avoidance of injury. The specific strategies in relation to ethical issues involve: (1) obtaining the informants’ agreements without imposing on them through fully explaining the aims of the study and the role both they and the researcher would play; (2) informing the participants about the privacy and confidentiality rules of conducting the study; and (3) the researcher’s empathy and understanding toward any of the participants’ description in relation to the sensitive issue regarding the participants’ frustration and stress of the incapability of taking care of their children with developmental disabilities, which was very important to encourage the participants to share their real perceptions.

#### Formation of Initial Dimensions and Questionnaire

Based on the theoretical considerations discussed before, this study first conducted a semi-structured interview to form initial dimensions and a questionnaire of FQoL of CDD in China. Forty family members of CDD were recruited according to purposeful sampling strategy. They were: (1) chosen through special schools and rehabilitation centers, because only those who would like to spend time and money on their child’s education and rehabilitation would understand the impact of a CDD on their family; (2) a family member who took the main responsibility of accompanying and caring for their CDD for quite a long time; (3) able to orally express their ideas and feelings in relation to FQoL; and (4) willing to communicate with the researchers, as only those who trusted the researchers would reveal their authentic voices. The interview involved such questions as “how do you feel about your family relationships, and why?,” “which parts of your family life are satisfying for you and your family?,” “what are your family’s physical and spiritual needs for better family life satisfaction?,” and “what are the challenges and problems in relation to your family life satisfaction?” All interviews were recorded and transcribed in written forms. Constant comparative analysis (Fram, 2013) was used to group similarities and differences in family members’ descriptions in relation to basic characteristics of family life satisfaction.

The initial Chinese FQoL Questionnaire (Chinese FQoL-Q) involves 10 dimensions: (1) physical health, i.e., physical conditions of family members; (2) mental health, i.e., psychological and emotional characteristics of the family; (3) family communication, i.e., the interpersonal relationship between family members; (4) financial situation, i.e., family income and expenditure; (5) leisure life, i.e., the degree and opportunity of family involvement in leisure activities; (6) career development, i.e., career development of family members; (7) parenting, i.e., the way and content of family education; (8) relationship with others, i.e., characteristics of the relationship between family members and others extended family members; (9) professional support, i.e., the family receives relevant support from the government, the disabled persons’ federation, and other departments; and (10) support from others, i.e., emotional and daily support from others or other families with CDD (Table 1).

**TABLE 1:** Categories and specific descriptions of parents with CDD.

**Category**	**Description**	**Class**	**Description**
PH	Good appetite; no medicine; Weariless; no disease	PS	Government financial support; schooling security; medical and rehabilitation support; social support such as a volunteer foundation; teachers’ help with children
MH	Emotional stability; optimistic about life; sense of security; psychological happiness; contentment	LA	Families play together; families have the opportunity to travel; have fun
FC	Family reunion; amity; discuss problems together; struggle together; respect and understanding between husband and wife; family unity; tolerance; harmony; no quarrel	CD	Smooth work; have a satisfactory job; find fun at work; able to work; achievements in work
FS	Suitable living environment; income can cover expenses; able to afford to see a doctor; basic food and shelter is ensured; no need to worry about food or clothing	PA	Know children’s school teachers; care about children’s studies; children get along with others; children are independent; pay attention to children’s upbringing; focus on the educational resources for children’s growth
SO	Emotional support and understanding; help with daily cooking; help from the elderly at home	RO	Respect; acceptance; good relationship

*PH, physical health; MH, mental health; FC, family communication; FS, financial situation; SO, support from others; PS, professional support; LA, leisure activities; CD, career development; PA, parenting; RO, relationship with others.*

The initial Chinese FQoL-Q contains 72 items that were constructed based on the two more widely used Western tools (the Beach Center FQoL-S and the International FQoL-S). For example, under the dimension of Parenting in the initial Chinese FQoL-Q, the description of the item “*My family helps children learn to be independent*” referenced to descriptions of items under Parenting in the Beach Center FQoL-S.

The 10 dimensions and 72 items on the initial Chinese FQoL-Q are not only consistent with Zuna et al. (2009) systematic, performance, individual-member, and family unit concepts, but also reflects the three themes: emphasizing family members’ subjective impressions of family life satisfaction, meeting individual family member needs, and taking the whole family as a unit to evaluate FQoL (Zuna et al., 2010). It was a self-reported questionnaire in which each item scored from 1 to 5, the highest score referring to the most consistent opinion toward QoL. All items were randomly arranged for further scrutiny.

#### Data Collection

The data were collected through special schools and educational rehabilitation institutions. The questionnaire was distributed along with the informed consent form. To ensure effective answers to the questionnaire, parents with a poor educational background or those who had difficulty in filling in the questionnaire were supported by the researchers (offering oral explanations) to complete the questionnaire.

#### Data Analysis

The data were input into SPSS and randomly divided into three groups: Sample A (*n* = 117) for item analysis, Sample B (*n* = 323) for exploratory factor analysis (EFA), and Sample C (*n* = 405) for confirmatory factor analysis (CFA) and reliability test.

Regarding item analysis of Sample A, extreme group comparison method was used (Wu, 2010) to determine the appropriateness of the items in the Chinese FQoL-Q.

The EFA was conducted for Sample B according to the principal component analysis for factor extraction. First, the polychoric correlations, the Kaiser–Meyer–Olkin (KMO), and Bartlett test were conducted to confirm whether the data were appropriate for EFA. Second, both orthogonal rotation and oblique rotation methods were used in order to identify an appropriate factor rotation method, and at last orthogonal varimax rotation was used because of similar results from the two methods, in terms of factors numbers and items (Kieffer, 1998). Third, parallel analysis was conducted to determine whether a factor should be kept or deleted (Kong and Zhang, 2007).

The CFA was carried out for Sample C by AMOS22.0. The Maximum Likelihood method is widely used to estimate model identification and analysis, with a premise that the sample data conforms to the multivariate normal distribution (Wu, 2009). Both first-order and second-order of CFA were used to judge whether a factor model of the Chinese FQoL-Q met psychological standards, according to requirements that χ^2^/df is between 1 and 3; standardized root mean square residual (SRMR) and root mean square error of approximation (RMSEA) are less than 0.08; comparative-fit index (CFI) and incremental fit index (IFI) are greater than 0.90; and parsimony normed fit index (PNFI) and parsimony comparative fit index (PCFI) are greater than 0.50 (Wu, 2009, pp. 44–50).

## Results

### Item Analysis

Extreme group comparison method for Sample A was used to determine the appropriateness of items in the Chinese FQoL-Q. On the basis of reverse scoring of reverse items, the scores of all items in the questionnaire were added to get a total score. The total scores were arranged from high to low, taking the top 27% scores as the group of high scores (Group 1) and the bottom 27% scores as the group of low scores (Group 2), according to the operating procedure of extreme group comparison method of item analysis (Qiu, 2013, p. 315). An independent sample T test was conducted using the scores of all items between Group 1 and Group 2. It was found that there was no significant difference in scores of items 4, 48, and 27, indicating that the discrimination degree of these three items is not enough. The three items were deleted, and sixty-nine items were left for further test.

### Exploratory Factor Analysis

Sample B was used for EFA in order to estimate any underlying factors and attach meaning to those factors of the Chinese FQoL Questionnaire. Before EFA, polychoric correlations was adopted to test correlations between the sixty-nine items. As a result, two items were deleted (item 7: “*Improving family economic condition is very crucial*” and item 9: “*My family member would quit their job for the sake of taking care of the kids*”). The reason is that the two items had low correlation with the rest of the items (less than 0.3) and were inappropriate for EFA (Wu, 2010, p. 206). The remaining 67 items were suitable for EFA: the KMO is 0.93 and the Bartlett’s Test is significant (*p* < 0.00, df = 2211).

The EFA was conducted with the remaining 67 items according to the principal components analysis for factor extraction (Wu, 2010, p. 199). Both orthogonal varimax rotation and oblique promax rotation were adopted for factor rotation (Kieffer, 1998). After conducting EFA many times, seven factors (35 items) were determined, and the same results were produced from orthogonal varimax rotation and oblique promax rotation.

**FIGURE 1:**
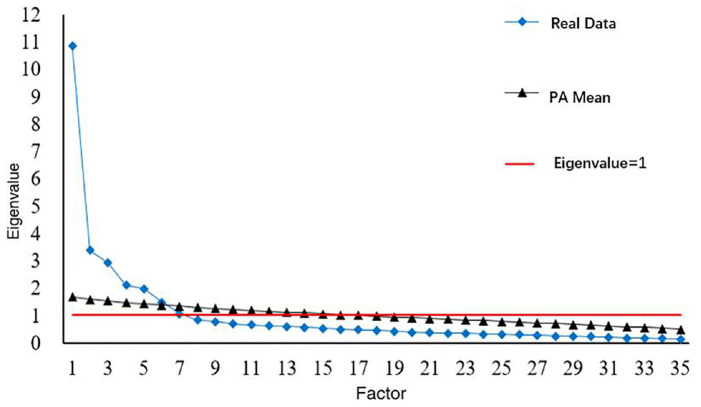
Plot of actual versus randomly generated eigenvalues.

In order to confirm the appropriateness of the seven factors, the Monte Carlo PCA for Parallel Analysis (Watkins, 2000) was adopted, as parallel analysis is objective and rigorous when determining whether a factor should be kept or deleted (Kong and Zhang, 2007). It was found that the first six factors should be kept as their eigenvalues from the real data correlation matrix were greater than the average eigenvalues from the random correlation matrices. As the eigenvalue of the seventh factor was only slightly lower than the seventh random eigenvalue (Figure 1), and the first six factors could explain only 62.08% of the variance of the Chinese FQoL, the seventh factor was also selected. The seven factors could explain 67.89% of the variance of the Chinese FQoL, and were identified as economy and leisure (f1), physical and mental health (f2), parenting (f3), family communication (f4), support from others (f5), professional support (f6), and career development (f7) (Table 2). The EFA yielded seven factors with 35 items, and the loading values ranged from 0.55 to 0.88 (Table 3).

**TABLE 2:** Characteristic value and variance contribution rate of FQL for children with developmental disabilities.

	**EL**	**PM**	**PA**	**FC**	**SO**	**PS**	**CD**	**Total variation (%)**
Eigenvalue after rotation	4.47	4.13	3.61	3.42	3.26	2.85	2.03	
Variance contribution rate (%) after rotation	12.75	11.80	10.32	9.76	9.31	8.15	5.81	67.89

*EL, economy and leisure; PM, physical and mental health; PA, parenting; FC, family communication; SO, support from others; PS, professional support; CD, career development.*

**TABLE 3:** Structure and Indexes of FQL for children with developmental disabilities.

**Dimension**	**Item**	**Item Description**	**Loading value**	**Communality**
EL (f1)	b53	All family members can participate in family leisure activities	0.83	0.77
	b60	My family will actively engage in leisure activities	0.84	0.79
	b17	My family has sufficient opportunities to participate in leisure activities	0.81	0.7
	b57	I am satisfied with how relaxed my family members are	0.73	0.64
	b6	My family can make ends meet	0.59	0.62
	b28	My family members have convenient transportation tools to get where they want to go	0.64	0.6
	b39	My family has a suitable living environment	0.55	0.58
PM (f2)	b1	For nearly a week, my family members have been in good health without any discomfort	0.74	0.6
	b24	For nearly a week, my family has been emotionally stable	0.78	0.72
	b35	For nearly a week, my family has been upbeat about life	0.72	0.72
	b46	For nearly a week, my family has been feeling safe	0.67	0.7
	b64	For nearly a week, my family members have been sleeping very wel	0.76	0.7
	b72	For nearly a week, my family’s appetite has been very good	0.76	0.7
PA (f3)	b16	My family develops skills to prepare children for life in the future	0.76	0.72
	b10	My family focuses on thinking about children’s future	0.76	0.68
	b32	My family helps children learn to be independent	0.85	0.79
	b43	My family teaches children how to get along with others	0.83	0.8
	b55	My family helps children finish schoolwork	0.7	0.64
FC (f4)	b3	My family members help each other	0.73	0.68
	b22	My family members respect each other’s hobbies and personal space	0.71	0.58
	b50	My family members will fight together for the future of our family	0.7	0.59
	b52	I’m happy with the relationships in my family	0.73	0.63
	b70	My family members trust each other	0.7	0.63
SO (f5)	b26	Relatives help with my family’s daily routine, such as shopping and taking care of the family	0.76	0.64
	b37	Relatives provide emotional support for my family, such as encouragement and listening	0.66	0.58
	b49	Neighbors help with my family’s daily routine, such as shopping and taking care of the family	0.83	0.73
	b67	Friends help with my family’s daily routine, such as shopping and taking care of the family	0.82	0.76
	b20	Friends provide emotional support for my family, such as encouragement and listening	0.7	0.63
PS (f6)	b29	My family can receive social support from foundations, non-profit organizations, volunteers, and others	0.75	0.62
	b18	My family can get financial support from the government (e.g., the civil affairs bureau and disabled persons’ federation) for their children	0.85	0.74
	b8	My family can receive relevant medical and rehabilitation support from the government (such as the civil affairs bureau and disabled persons’ federation).	0.88	0.79
	b30	I am satisfied with the professional support services my family receives	0.77	0.69
CD (f7)	b31	My family will pursue work or study that they love	0.65	0.6
	b42	My family is doing well at work	0.67	0.75
	b59	My family members are satisfied with their current jobs	0.66	0.67

*EL, economy and leisure; PM, physical and mental health; PA, parenting; FC, family communication; SO, support from others; PS, professional support; CD, career development.*

### Confirmatory Factor Analysis

The core of CFA involves model identification, analysis, and evaluation. The Maximum Likelihood method is widely adopted to estimate model identification and analysis, with a premise that the sample data conform to the multivariate normal distribution (Wu, 2009, p. 24). The normal distribution test of sample C was carried out by adopting QQ diagram in SPSS, instead of Z test. The reason is that the Z test method, based on kurtosis and skewness, is easily affected by the number of samples; when the sample size exceeds 100, it is easy to misjudge the data as non-normal (Qiu, 2013, pp. 105–107). It was found that the points in the normal QQ diagram of 35 items fall on or near the 45 degree reference line, indicating that the data of 35 items conform to multivariate normal distribution and that Maximum Likelihood can be used for CFA.

The fitting judgment of CFA results mainly depends on absolute fit indices, incremental fit indices, and parsimony fit indices (Hooper et al., 2008). It is recommended to consider a variety of fit indices so that the weakness of a particular index is counteracted by the strength of another (Dardas and Ahmad, 2014). Taking into account the frequency of utilization and stability of indices (Wu, 2009, pp. 40–52), χ^2^/df, SRMR, and RMSEA are selected for the absolute fit index. IFI and CFI are selected for the incremental fit index. PNFI and PCFI are selected for the parsimony fit index.

**FIGURE 2:**
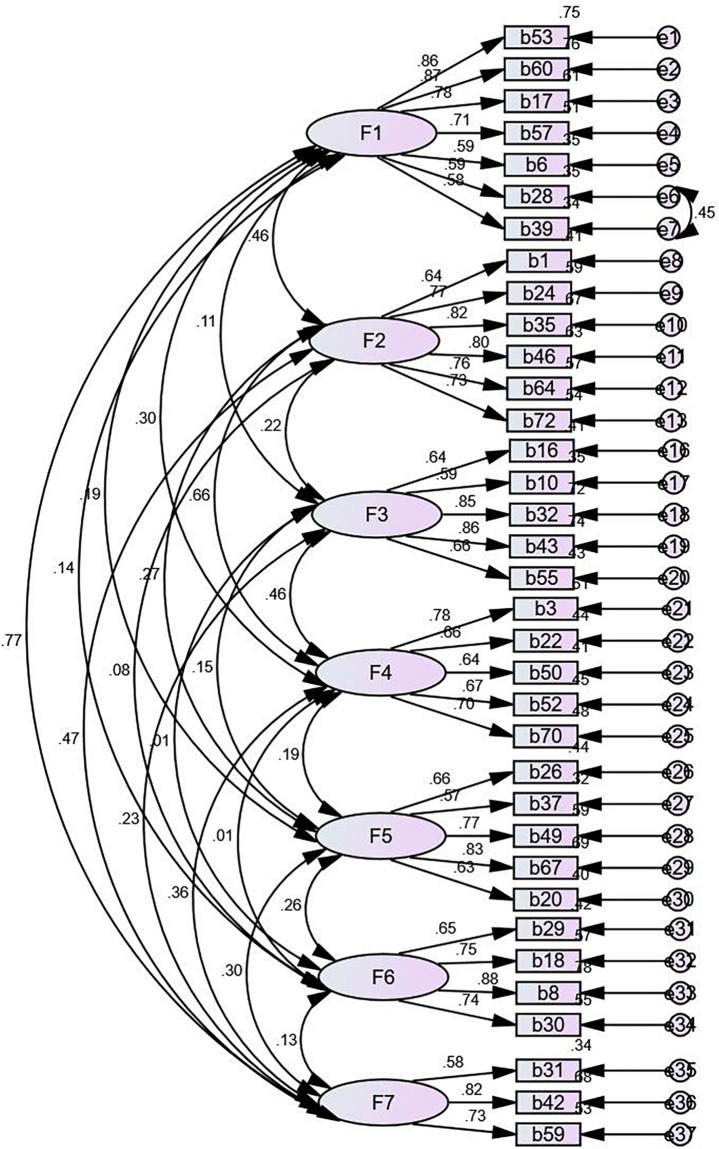
First-order confirmatory factor analysis.

**FIGURE 3:**
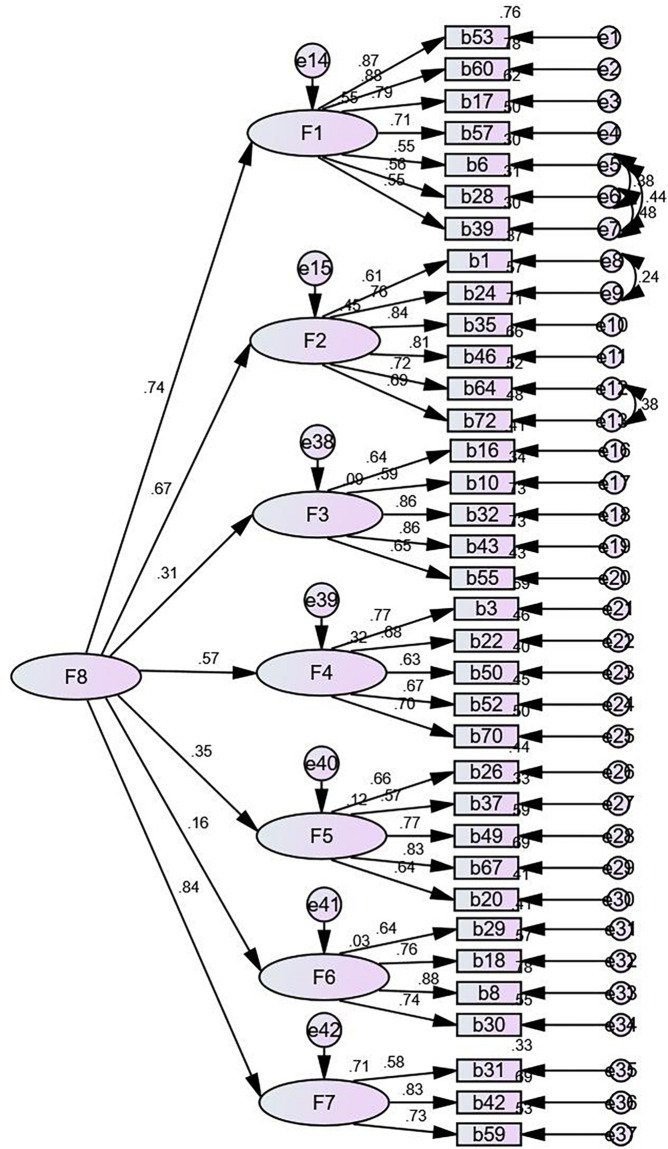
Second-order confirmatory factor analysis.

In the first-order of CFA, χ^2^/df is 2.21, SRMR is 0.06, RMSEA is 0.06, IFI is 0.91, CFI is 0.91, PNFI is 0.76, and PCFI is 0.82, which meets the requirements that χ^2^/df is between 1 and 3, SRMR and RMSEA are less than 0.08, CFI and IFI are greater than 0.90, and PNFI and PCFI are greater than 0.50 (Wu, 2009, pp. 44–50). However, there were middle to high correlations between several factors (e.g., Economy and leisure and career development) in the first-order of CFA (Figure 2). A second-order of CFA was conducted to explore whether the seven factors in the first order of CFA tested the same quality (Figure 3). In the second order of CFA, χ^2^/df is 2.19, SRMR is 0.07, RMSEA is 0.05, IFI is 0.91, CFI is 0.91, PNFI is 0.77, and PCFI is 0.83. Meanwhile, all factor weights are significant. Therefore, the CFA demonstrates that the seven-factors model of the Chinese FQoL Questionnaire for CDD meets psychological standards and has a reliable construct validity.

### Reliability Test

On the basis of CFA, Cronbach’s alphas of the Chinese FQoL-Q and its dimensions were tested according to Sample C (Table 4). The Cronbach’s alpha of the Chinese FQoL-Q is 0.89, and Cronbach’s alpha of each dimension is above 0.70, which meets psychological standards (Wu, 2010, p. 244). The result shows that the seven-factor model of the Chinese FQoL-Q for CDD has good reliability.

**TABLE 4:** Reliability Coefficient of Family Quality of Life and Each Dimension for children with developmental disabilities.

	**Total**	**EL**	**PM**	**PA**	**FC**	**SO**	**PS**	**CD**
Cronbach’s α	0.89	0.89	0.89	0.84	0.82	0.82	0.84	0.75

*EL, economy and leisure; PM, physical and mental health; PA, parenting; FC, family communication; SO, support from others; PS, professional support; CD, career development.*

## Discussion

Improving QoL of families of CDD is not only conducive to CDD’s educational development and rehabilitation, but also helpful in improving the living conditions of their family members. However, research on FQoL of CDD in China mainly focuses on investigating the *status quo* by borrowing the Western scales (Hu and Wang, 2012) or using individual interviews (Luo, 2014; Li, 2016). It is crucial to construct a FQoL scale for families of CDD within the context of China.

The initial construction of the Chinese FQoL-Q was derived from interviews with 40 family members of CDD, which was based on the theoretical consideration of this study. The initial Chinese FQoL-Q contains 10 dimensions and 72 items, focusing on family members’ descriptions of characteristics in relation to FQoL. It was sent to a sample of 846 family members’ of CDD, and survey data were collected to conduct item analysis, EFA, and CFA and reliability test in order to explore the factor structure and psychometric properties of the Chinese FQoL-Q for CDD against the background of Chinese culture.

It was found that the final Chinese FQoL-Q involves seven factors: economy and leisure, physical and mental health, parenting, family communication, support from others, professional support, and career development. There are 35 items that were appropriate in terms of statistical indicator and specific content. During CFA, this study conducted model revision according to modification indices to improve model fit. Specifically, in the second-order of CFA, covariances between the errors for b6 and b28 and b39 were added, and the three items could be further improved and tested in future.

The dimensions and items on the final Chinese FQoL-Q reflect family members’ of CDD evaluations of their whole families’ life satisfaction that was subjectively presented, and their individual family member’s needs were also considered (Zuna et al., 2010). Meanwhile, the dimensions and items on the final Chinese FQoL-Q are conceptually consistent with Zuna et al.s’ (2009) systematic, performance, individual-member, and family unit concepts.

The dimensions and items on the Chinese FQoL-Q reflect the challenges and dilemmas that the Chinese families of CDD encountered, i.e., the dimensions of economy and leisure, parenting, professional support, and support from others. As discussed before, the Chinese families of CDD are struggling with financial difficulty, poor quality of professional rehabilitation, and a shortage of time and energy in relation to taking care of their kids (Huang and Liu, 2006; Chen and Chen, 2016). Their family QoL would be largely improved if provided with these supports.

The Chinese FQoL-Q shares several similarities with the three most important disability-related measures of FQoL in the West. For example, both the Chinese FQoL-Q and the Beacher Center FQoL-S have similar dimensions and items in relation to Parenting, Family Communication or Interaction, Emotional Well-being, or Physical and Mental Health. The Chinese FQoL-Q shares similar dimensions with the International FQoL-S, in terms of dimensions and items in relation to careers and support from others. The dimension of Physical and Mental Health and its items on the Chinese FQoL-Q were similar to the dimensions and items in relation to Emotional Well-being and Physical/material Well-being on the Latin American FQoL-S. The similarities among these tools demonstrates the fact that the evaluation of families’ QoL of CDD share common features across different cultures, in terms of physical and psychological conditions, interpersonal relationships, and material and economic conditions.

The specific differences between the Chinese FQoL-Q and the Beach Center FQoL-S and the International FQoL-S will be discussed, because the two Western tools are internationally used in countries and different cultural contexts (Samuel et al., 2012), while the Latin American FQoL-S was specifically tailored for the high unemployment rate and poverty status that exists in Latin America.

**FIGURE 4:**
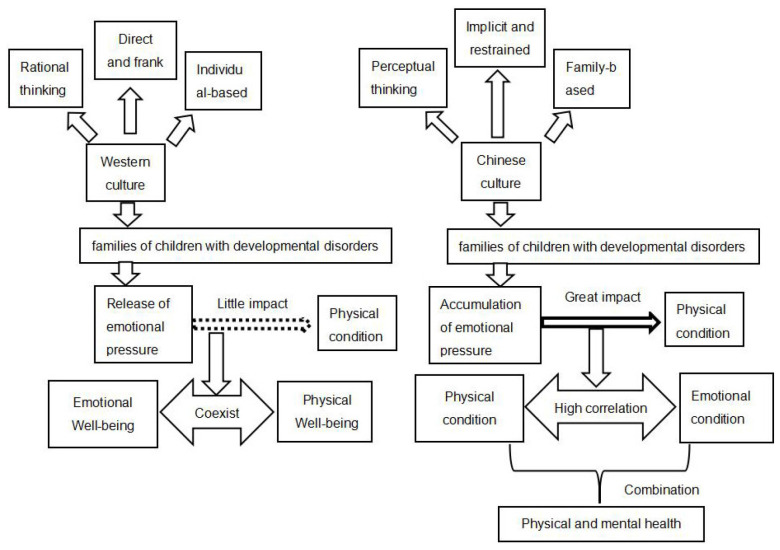
Physical and mental health and emotional well-being and physical well-being dimensions under the cultural differences between China and the West.

First, the Chinese FQoL-Q only contains a dimension of Physical and Mental health that is similar to both the dimensions of Emotional Well-being and Physical Well-being on the Beach Center FQoL-S. The Chinese family based and perceptual thinking are different from the Western individual-based and rational thinking, probably incurring more sources of pressure for Chinese families with CDD. The fact that individual interests in China are subordinate to their family, group, and the state (Xu et al., 2018) have made the Chinese parents more concerned with their children’ disabilities and the whole families’ difficulties, probably leading to serious emotional distress. This situation may exacerbate as their children and families are given priority, instead of their own physical and emotional well-being. Whereas the Western families probably tend to distinguish their children’s disabilities and family difficulties from their own personal goals and prioritize their own goals. That is, the Chinese way of thinking tends to be perceptual thinking leading to Chinese parents being more likely to experience stress emotionally, whereas the Westerners mainly think rationally to face and solve problems and challenges caused by having CDD (Huo, 2018). Moreover, the Chinese tend to be reserved, introverted, and euphemistic, and are not good at expressing their emotions (Cheng, 2016) or forbearing their joys and sorrows (Liu, 2014). This is quite opposite to the Western culture that advocates a direct, frank, and natural way of expression through frequent exchanges between people (Yang, 2012). Compared to the West, Chinese families with CDD may not be good at releasing their emotional stress. As a result, negative emotions may build up and have a great adverse impact on physical health. This strengthens the connection between emotional state and physical health. Thus, physical health and mental health are combined into one dimension in the Chinese FQoL-Q due to the family based, perceptual thinking, and personality tendency of the Chinese people (Figure 4).

Second, the Chinese FQoL-Q only contains a dimension of Economy and Leisure that is similar to both the dimensions of Financial Well-being and Leisure and Recreation on the International FQoL-S. The concept of leisure in China is regarded as being opposed to work and jobs, whereas the Western leisure means idleness, free-time, and space that an individual could dominate on his or her own in order to realize personal interests, self-exploration, and self-satisfaction (Liu, 2014). Western leisure is regarded as the core indicator to examine human initiative and is the highest form of life in terms of rights, happiness and freedom, awe and belief, personal moral activities, and self-realization (Liu, 2014). However, influenced by Lao Tzu and Confucius, Chinese leisure is restricted by a realistic basis (i.e., economic level) and a focus on seeking personal inner peace (Toynbee, 1986). The main form of leisure for the majority of Chinese people is related to tourism (Liu, 2014). Only when a family has enough economic support will they consider leisure (Song, 2014). Specifically, most families with CDD in China encounter finance challenges for taking care of such children (Chen and Chen, 2016). Their leisure is bound to be related to whether their family economic conditions permit leisure or not (Luo, 2014). That is why Economy and Leisure in the Chinese FQoL-Q constitutes one dimension.

Third, the dimension of Community Interaction on the International FQoL-S describes the quality of communication and relationships between the family members of CDD and community members such as neighbors (Isaacs et al., 2007). However, this dimension was deleted in factor analysis of this study. It means that Chinese families with CDD do not consider communication with others as an important indicator for satisfying their families’ basic needs. As discussed above, the Chinese families with CDD who are influenced by traditional Chinese culture tend to first consider the needs of their CDD and difficulties their family encounter because of their children’s deficits, and they are willing to sacrifice their interpersonal communication time to prioritize the needs of their children and families (Huo, 2018). On the contrary, Western society emphasizes that the realization of individual needs is superior to the family, and most families with CDD in the West would not ignore their own interpersonal needs just because of their children’s disabilities. Moreover, the general republic’s acceptance of disabilities is a concern in China (Xu et al., 2018). Domestic research has indicated that a negative attitude toward children with disabilities and their families has a great impact on family members’ enthusiasm for interpersonal communication (Huang and Shen, 2017). That is, Chinese parents would be unwilling to walk into a community and keep in contact with community members. Furthermore, interpersonal communication in China usually involves gossip, e.g., sharing their children’s education and development in order to make comparisons. This makes them feel humiliated or trigger feelings of inferiority, so they tend to avoid socializing with persons in the community (Huang and Shen, 2017). This is quite opposite to the Western culture in relation to positive views of family members with CDD toward community interactions. As indicated by Brown and colleagues (2003), Canadian parents tended to participate in community affairs and interact with community members because they believed that through community interactions they could get more natural support, as well as promote government policy-making beneficial to their children and their families.

Fourth, the dimension of Parenting in this study involves “*developing skills for future life, learning to be independent, getting along with others, and completing their studies*.” In addition to this, the Beach Center FQoL-S also involved “*paying attention to the unique needs of the child, getting to know the child’s friends, helping the child make decisions, and respecting the child’s personality and rights*.” The ideal for Chinese parents with CDD appeared to be pragmatism that mainly focused on promoting the child’s skills in relation to learning, interpersonal communication, independence, and skills needed for future life, whereas Western parents tended to concern themselves more with the equality of their children (Wang, 2014). Influenced by individualism, Western people tend to respect individual rights and yearn for freedom, equality, and democracy (Wang, 2014). That is why in the West, parents respect their children’s personalized needs, advocate for equality for their children, and thus help them make decisions instead of making decisions for their children. In contrast, collectivism in current China, influenced by Confucian philosophy, attaches great importance to the interests of family, society, and the state (Zheng, 2009). Under such circumstances, individual needs were ignored. This is reflected in the parenting dimension of this study, showing that the Chinese parents do not focus on the unique needs of children, but emphasizes the authority of family (parents).

Fifth, in the dimension of Physical and Mental health, Chinese parents were mainly concerned with sleep status, physical condition, appetite, sense of security, and emotional stability, whereas the Beach Center FQoL-S emphasizes whether the family member could have regular checkups, emotional release, and the capacity to do what they are interested in. Such differences probably lie in that the Chinese traditional culture focuses more on the stability of the *status quo* of personal life and lacks initiative, while the Western culture advocates initiative and exploration (Liu, 2014). Moreover, because of collectivism, the Chinese FQoL-Q did not attach great significance to individualized-related items as indicated in the West, such as “r*elease of personal emotions”* and *“doing what you are interested in*.”

Sixth, the Chinese parents tend to seek “medical, rehabilitation, financial, and social support,” as indicated in the dimension of Professional Support. Such general demands from Chinese parents are quite different from the West, where demands are meticulous and clear, such as “*having good interpersonal relationships with professional service personnel,” “children with disabilities getting support from making friends,”* and *“obtaining support from participating in activities at school or family.”* A possible reason for this might be that special education in China starts relatively later than the West (Lu, 2017); the families’ idea of special education lags behind that of Western countries (Lu, 2017; Zhang and Lin, 2018).

## Limitations and Suggestions

First, Chinese culture is a complicated system that includes many sub-cultural systems, e.g., the majority “Han” and the remote minorities. It should be further discussed to what extent that the factor structure of the Chinese FQoL-Q for CDD differs among different sub-cultural systems. Further research should focus on exploring the sub-cultural systems in detail and then presenting a comprehensive interpretation of the cultural impact on FQoL for CDD.

Second, the participants of this study mostly involved parents of children with autism and intellectual disabilities, which are the two most common types of developmental disabilities. However, the range of developmental disabilities includes other types of disabilities, such as emotional behavior disorder, and different types of disabilities have different diagnosis standards and levels of severity. Future research should explore whether the Chinese FQoL-Q could be applied to different types of disabilities and levels of severity in relation to developmental disabilities.

Third, it is worth noting the dynamics of the Chinese FQoL-Q. Because education and rehabilitation for people with disabilities is changing dramatically, parents’ understanding of and need for professional support services would change, as well as their perception in relation to family quality of life. The construction of the factor structure and index system of family quality of life is an ongoing and evolving process.

The Chinese FQoL-Q reflects traditional Chinese culture and parents’ perception of education or rehabilitation for their CDD in China. It is reliable and valid for assessing the quality of life for family members of CDD.

## Data Availability Statement

All datasets generated for this study are included in the article/supplementary material.

## Ethics Statement

The studies involving human participants were reviewed and approved by Chongqing Normal University and Guizhou Institute of Engineering and Applied Technology. The patients/participants provided their written informed consent to participate in this study.

## Author Contributions

All authors listed have made a substantial, direct and intellectual contribution to the work, and approved it for publication.

## Conflict of Interest

The authors declare that the research was conducted in the absence of any commercial or financial relationships that could be construed as a potential conflict of interest.
